# CT appearances, patterns of progression, and follow-up of COVID-19: evaluation on thin-section CT

**DOI:** 10.1186/s13244-021-01019-0

**Published:** 2021-06-10

**Authors:** Chun-Shuang Guan, Zhi-Bin Lv, Jing-Jing Li, Yan-Ni Du, Hui Chen, Tao Cui, Ning Guo, Bu-Dong Chen, Ru-Ming Xie

**Affiliations:** 1 Department of Radiology, Beijing Ditan Hospital, Capital Medical University, No. 8 Jingshun East Street, Chaoyang District, Beijing, China; 2 Department of Clinical Research, Shukun (Beijing) Technology Co., Ltd., Jinhui Bd, Qiyang Rd, Chaoyang District, Beijing, China

**Keywords:** COVID-19, Pneumonia, Infectious disease medicine, Multidetector computed tomography

## Abstract

**Background:**

To retrospectively analyze CT appearances and progression pattern of COVID-19 during hospitalization, and analyze imaging findings of follow-up on thin-section CT.

**Methods:**

CT findings of 69 patients with COVID-19 were evaluated on initial CT, peak CT, and pre-discharge CT. CT pattern were divided into four types on CT progression. Lesion percentage of pulmonary lobe (lobe score) was graded. Correlation analysis was made between scores and intervals. 53 patients were followed up by CT.

**Results:**

Among 69 patients, 33.3% exhibited improvement pattern, 65.2% peak pattern, 1.5% deterioration pattern, and 0% fluctuation pattern. The lobe scores were positively correlated with most of intervals. It was more common to observe consolidation, pleural thickening and pleural effusion on the peak CT, and irregular line and reticulation on pre-discharge CT. The peak-initial interval were shortened when the initial CT with consolidation and pleural thickening. The intervals were extended when the irregular lines appeared on peak CT and reticulation on pre-discharge CT. Among 53 follow-up patients, 37.7% showed normal chest CT, and 62.3% showed viral pneumonia remained that mainly included GGO (100.0%) and irregular lines (33.3%).

**Conclusions:**

COVID-19 displayed different appearances on CT as progressing. The peak pattern was the most common progression pattern. The CT appearances showed closely related to the intervals. The COVID-19 pneumonia can be remained or completely absorbed on CT with follow-up.

## Key points

- The most progression pattern was peak pattern.
- Lobe score showed a positive correlation with intervals
- Intervals were more extended as irregular lines and reticulation on CT.
- COVID-19 can be remained or completely absorbed on chest CT with follow-up.

## Background

Coronavirus disease 2019 (COVID-19), which is caused by severe acute respiratory syndrome-associated coronavirus 2 (SARS-CoV-2). COVID-19 is first reported in Wuhan, China on December 31, 2019 [1], which spreads and progresses very quickly. By March 11 2020, the World Health Organization (WHO) classified the outbreak as a pandemic [2]. COVID-19 is still diffused across different countries at present. As of April 10, 2021, there were 133,552,774 confirmed cases and 2,894,295 deaths worldwide [3]. At present, the United States is the country with the largest number of confirmed patients (30,615,849 confirmed cases, and 553,801 deaths), following by Brazil (13,193,205 confirmed cases, and 340,776 deaths) and India (13,060,542 confirmed cases, and 167,642 deaths) [4]. Computed tomography (CT) scanning was one of the important examinations for a diagnosis of COVID-19 [5, 6], and the CT images change rapidly in COVID-19. The patients show different findings of chest CT in different periods, [7–10]. Ground-glass opacity (GGO) is presented as the main manifestation in the early stage, crazy-paving pattern mainly in the progressive stage, consolidation mainly in the peak stage, the consolidation is gradually absorbed and fibrotic changes arise in the recovery stage [7–10]. To obtain better understanding of COVID-19 progression and follow-up changes on CT, we carried out the retrospective study on the CT appearances, pattern of progression and follow-up of discharged patients.

## Methods

### Materials

This study was approved by the Institutional Review Committee and the Ethics Committee of the institution (Beijing Ethics 2020 No. 022-01). Written informed consent for the retrospective analysis study was waived by the Institutional Review Board. All patients who were diagnosed as having SARS-CoV-2 by gene sequencing by fluorescence-based real-time reverse transcription-polymerase chain reaction (RT-PCR) were collected from January 12 to May 31, 2020. The inclusion criteria were: (1) adult patients (≥ 18 years old); (2) patient with positive findings of COVID-19 pneumonia on chest thin-section CT; (3) patients discharged from hospital, who met the discharge criteria, including the body temperature returning to normal for more than 3 days, the respiratory symptoms obviously improving, the lung imaging showing the acute exudative lesions obviously improving, and the nucleic acid test of respiratory tract samples such as sputum and nasopharyngeal swabs for two consecutive times was negative (the sampling time interval was at least 24 h). The exclusion criteria were: (1) patients without chest CT scans; (2) patients with other diffuse pulmonary disease in previous medical history or patients with other pathogens infection of clinical diagnosis; (3) patients without COVID-19 pneumonia on chest CT, that was, COVID-19 patients showed the normal chest CT; (4) patients remaining in hospital; (5) patients who did not return to the hospital for checks when collecting follow-up data. The clinical features (fever, cough, expectoration, chest tightness, dyspnea, runny nose, fatigue, hypogeusia, anosmia, diarrhea) and previous medical history were collected.

### Scanning equipment and scanning method

All patients underwent 16-slice spiral CT (Siemens, Sensation CT, Forchheim, Germany). All patients were supine with arms raised and held their breath at the end of inhalation during scanning. The scanning range was from the tip to the base of the lung. The slice thickness was 5.0 mm, and then the thin-section images with a slice thickness of 1.5 mm were reconstructed by a lung reconstruction algorithm. The tube voltage was 130 kV. The tube current was tube current modulation. The matrix was 512 × 512.

### Qualitative evaluation on chest CT

**Fig. 1:**
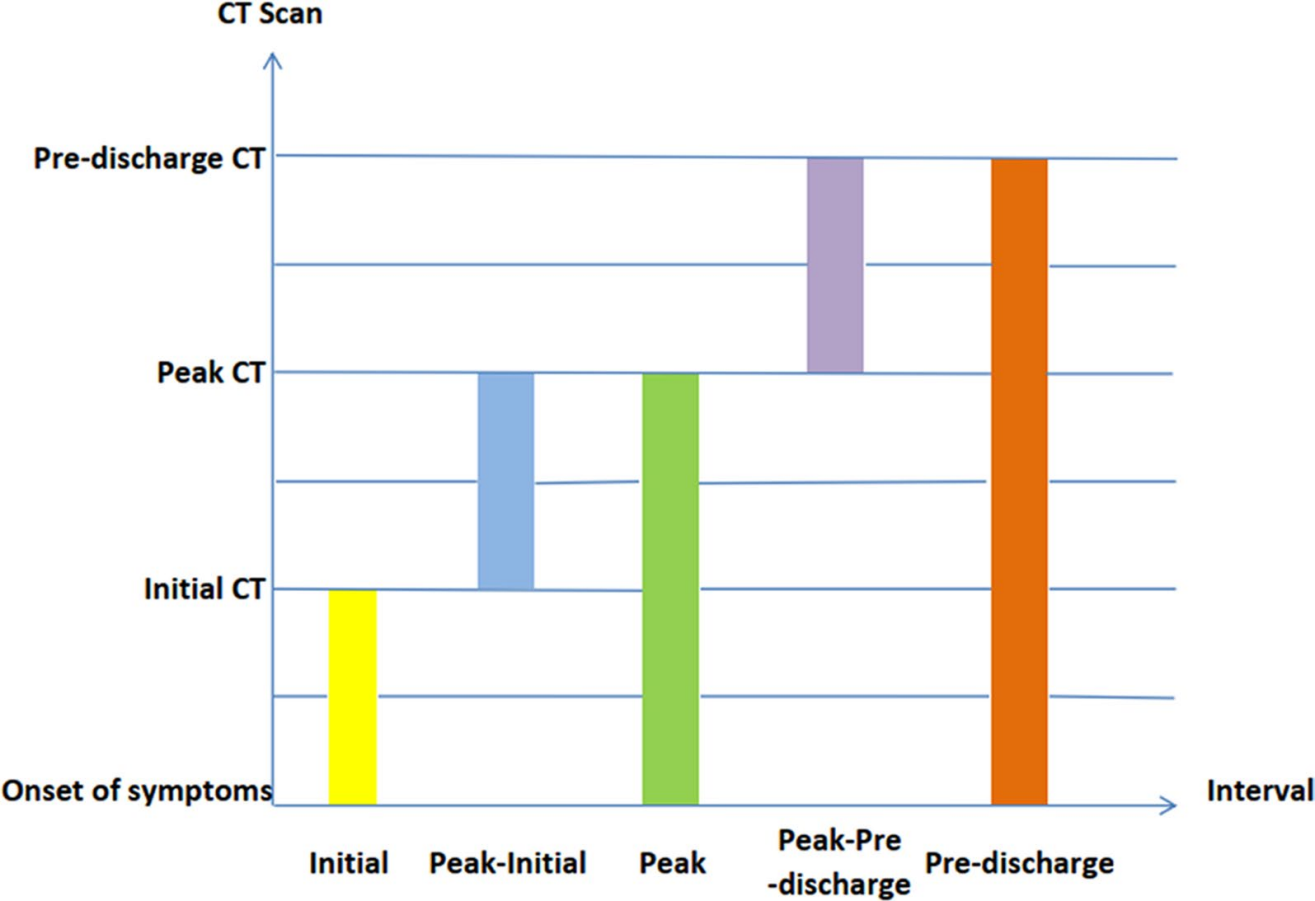
Schematic diagram of different time intervals

Two chest radiologists with 13 and 15 years of working experience independently evaluated the images on the Picture Archiving and Communication System (Carestream Health, Rochester, NY, US) and reached agreement through consensus. Initial CT was defined as first CT examination after the hospital visit. Peak CT was that with the largest size of lesion on CT or the highest attenuation of the density when no change in the size during COVID-19 progression. Pre-discharge CT was defined as the last CT examination before patients discharged from hospital. The time intervals included initial interval (interval between onset of symptoms and initial CT examination), peak interval (interval between onset of symptoms and peak CT examination), pre-discharge interval (interval between onset of symptoms and pre-discharge CT examination), peak-initial interval (interval between peak CT and initial CT examination), pre-discharge-peak interval (interval between pre-discharge CT and peak CT examination) (Fig. 1).

Based on CT progression during hospitalization, the CT patterns were divided into four types. Type I (improvement pattern) exhibited improving on CT. Type II (peak pattern) showed initial CT deteriorating to a peak level following by improvement. Type III (deterioration pattern) was defined as deteriorating on CT. Type IV (fluctuation pattern) showed fluctuation changes with at least two peaks on CT.

The lesion distributions on CT were divided into subpleural distribution and peribronchovascular bundle distribution. Subpleural area was defined as mainly distributing along the subpleural 1/3 region. Peribronchovascular bundle distribution was defined as mainly distributing around bronchovascular bundle.

CT appearances included GGO, consolidation, crazy-paving pattern, air bronchogram, irregular line, reticulation, pleural thickening, pleural effusion, nodule and lymphadenopathy. GGO referred to a fuzzy increase in lung density that does not cover blood vessels. Consolidation was defined as increased attenuation in density that covered blood vessels. Crazy-paving pattern referred to reticular shadow superimposed with GGO. Air bronchogram was defined as bronchus containing gas outlined by high attenuation of lung parenchyma. Irregular line referred to irregular linear shadow with thickness of 1-3mm, excluding interlobular septa thickening and intralobular lines. Reticulation was formed only by interlaced lines without GGO. Nodule referred to round shadows of high attenuation with clear or fuzzy edges, < 3.0 cm in diameter. Pleural effusion refers to abnormal fluid in pleural cavity. Lymphadenopathy was defined as lymph nodes with > 1.0 cm in short diameter [11].

### Quantitative evaluation on chest CT

Pulmonary lesions were scored according to the lesion size. The scoring criterion was defined as the percentage of lesions in one lobe (here termed lobe score). The lobes included upper, middle and lower lobes of right lung, and apicoposterior and anterior segments of left upper lobe, the upper and lower lingula segments of left upper lobe, and lower lobe of left lung. The lobe scores were as follows: 0 with no lesion in the lung, 1 with 0% < percentage (*P*) < 25%, score of 2 with 25% ≤ *P* < 50%, score of 3 with 50% ≤ *P* < 75%, score of 4 with *P* ≥ 75%. The total score of the whole lung was 1-24.

### Follow-up CT

With follow-up, when pneumonia lesions were completely absorbed on CT, the CT was taken as the follow-up CT. If there were pneumonia lesions remained on CT all the time, the last CT was taken as the follow-up CT. And the follow-up patients were divided into two groups, including absorption group and residual group. The absorption group referred to the complete absorption of viral pneumonia lesions on CT, and the residual group refers to the viral pneumonia lesions remaining on CT.

### Statistical analysis

Statistical analysis was performed using SPSS17.0 software (IBM, Armonk, NY, US). Continuous data were expressed as the mean and standard deviation or median (25th percentile, 75th percentile), and categorical data were expressed as the frequency. The distributions and imaging manifestations on initial CT, peak CT, and pre-discharge CT were analyzed by Chi-square test or Fisher's extract test. Analysis of repeated measures of variance and Friedman analysis were applied to the lobe scores on CT. Correlations were analyzed by multivariate linear regression. Absolute values of correlation coefficients (*r*) were graded as: 0.15–0.24, very low; 0.25–0.49, low; 0.50–0.69, moderate; 0.70–0.89, high; 0.90–1.00, very high. The analysis of variance and Mann-Whitney test were used to analyze the statistical differences of intervals on the imaging manifestations of initial, peak, and pre-discharge CTs. The follow-up CTs were analyzed by t test, Chi-square test, or Fisher's extract test. *p* value < 0.05 was considered statistically difference.

## Results

### Basic information of patients

This study collected 135 patients with COVID-19. Sixty-six patients were excluded. Sixty-nine patients were enrolled and analyzed retrospectively (37 females and 32 males, mean age 46.058 ± 15.641 years; range, 18–80 years). The 67 patients were evaluated on initial CTs because the two of 69 patients presented as the normal CT but showed the COVID-19 pneumonia on the peak CT and pre-discharge CT. All of the 69 patients were evaluated on the peak CT and pre-discharge CT. The most common clinical features were fever (76.8%,53/69), following cough (68.1%, 47/69), dry throat (29.0%, 20/69), runny nose (27.5%, 19/69), fatigue (27.5%, 19/69), headache (24.6%, 17/69), expectoration (21.7%, 15/69), chest tightness (13.0%,9/69), diarrhea (11.6%,8/69) dyspnea (7.2%,5/69), anosmia (8.7%,6/69), hypogeusia (4.35%,3/69). Some of the patients were accompanied by previous medical history, including hypertension (14.5%,10/69), heart disease (5.8%,4/69), diabetes mellitus (7.2%,5/69), and cerebrovascular disease (2.9%,2/69). The CT patterns of the 69 patients on CT showed that 23 (33.3%) were type I, 45 (65.2%) type II, one (1.5%) in Type III, and none Type IV.

**Fig. 2:**
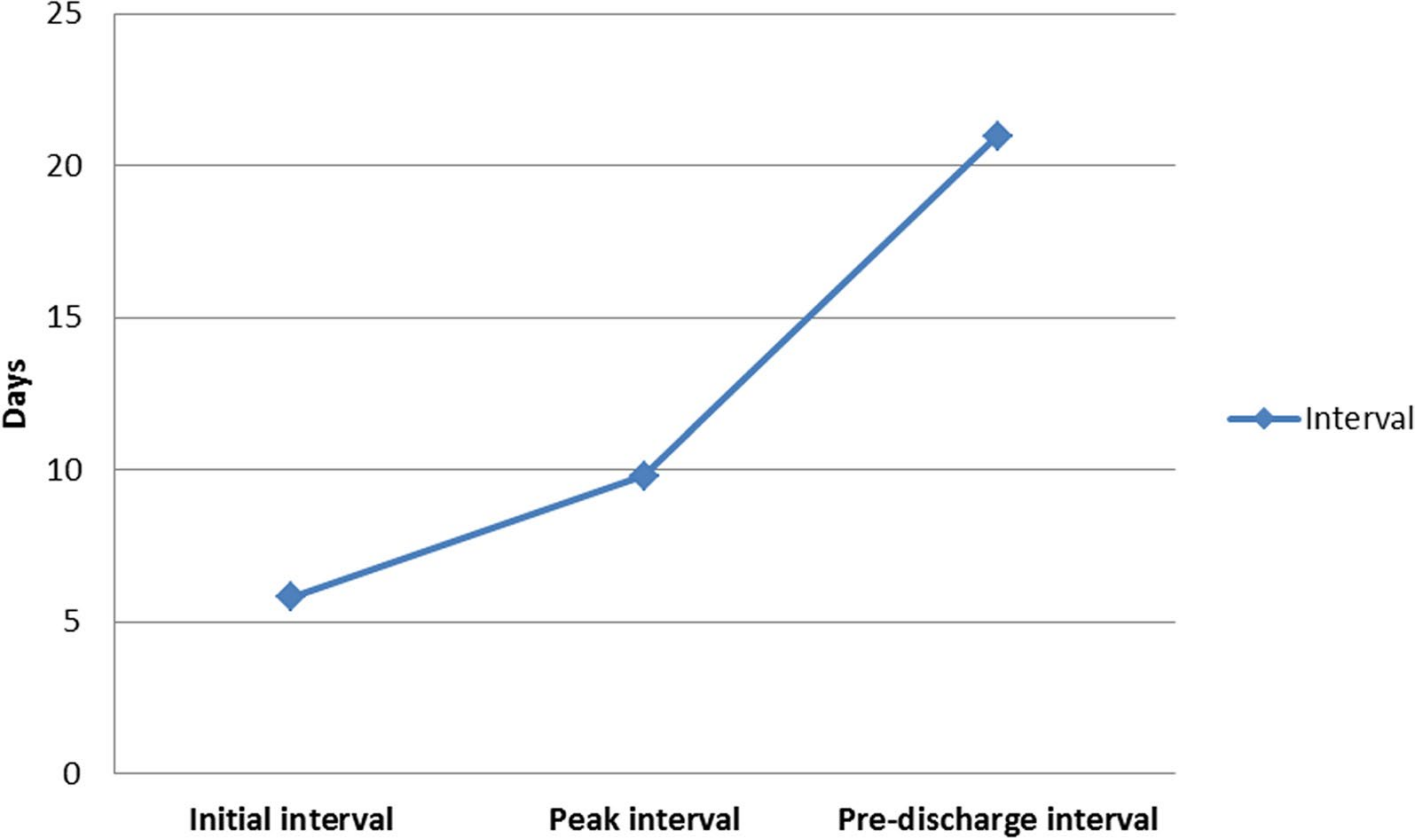
The intervals between the onset of symptoms and initial CT, peak CT, and pre-discharge CT

The intervals of the 69 patients were as follows: initial interval (5.81 ± 3.548 days), peak interval (9.80 ± 3.763 days), pre-discharge interval (20.97 ± 7.456 days), peak-initial interval (3.99 ± 3.281 days), pre-discharge-peak interval (11.19 ± 6.330 days). The peak-initial interval was significantly shorter than pre-discharge-peak interval (*p* < 0.001) (Fig. 2).

### Distribution

**Fig. 3:**
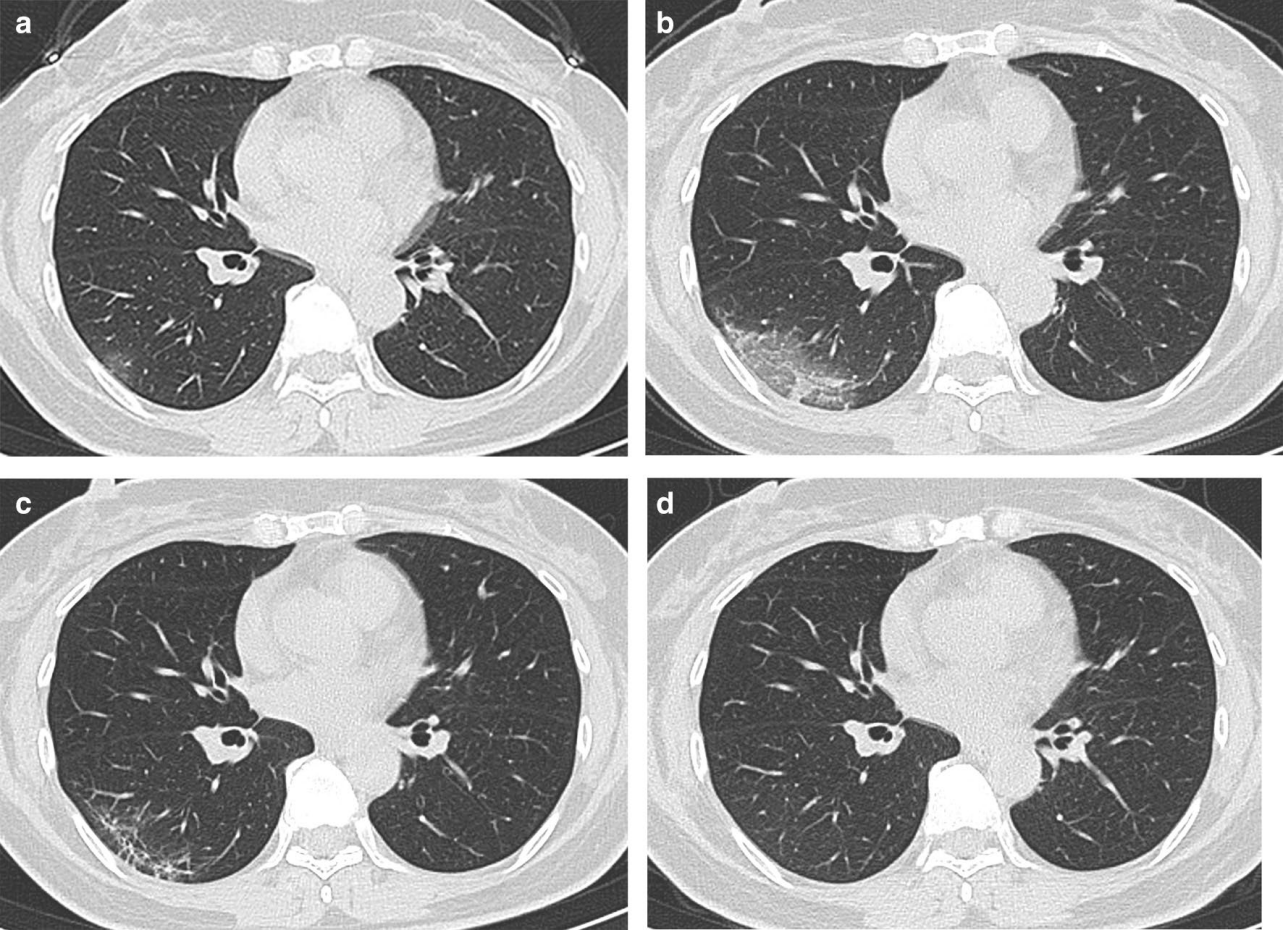
Female, 50-year-old, COVID-19. **a** The initial CT obtained 4 days from onset of symptoms shows the round ground-glass opacity around the subpleural area of the right lower lobe. **b** The peak CT obtained 6 days later shows the large patchy ground-glass opacity and a few of consolidation around the subpleural area of the right lower lobe. **c** The pre-discharge CT obtained 14 days later shows reticulation, irregular lines, and pleura thickening. **d**. Axial CT. The follow-up CT obtained 64 days later shows a normal chest CT

**Fig. 4:**
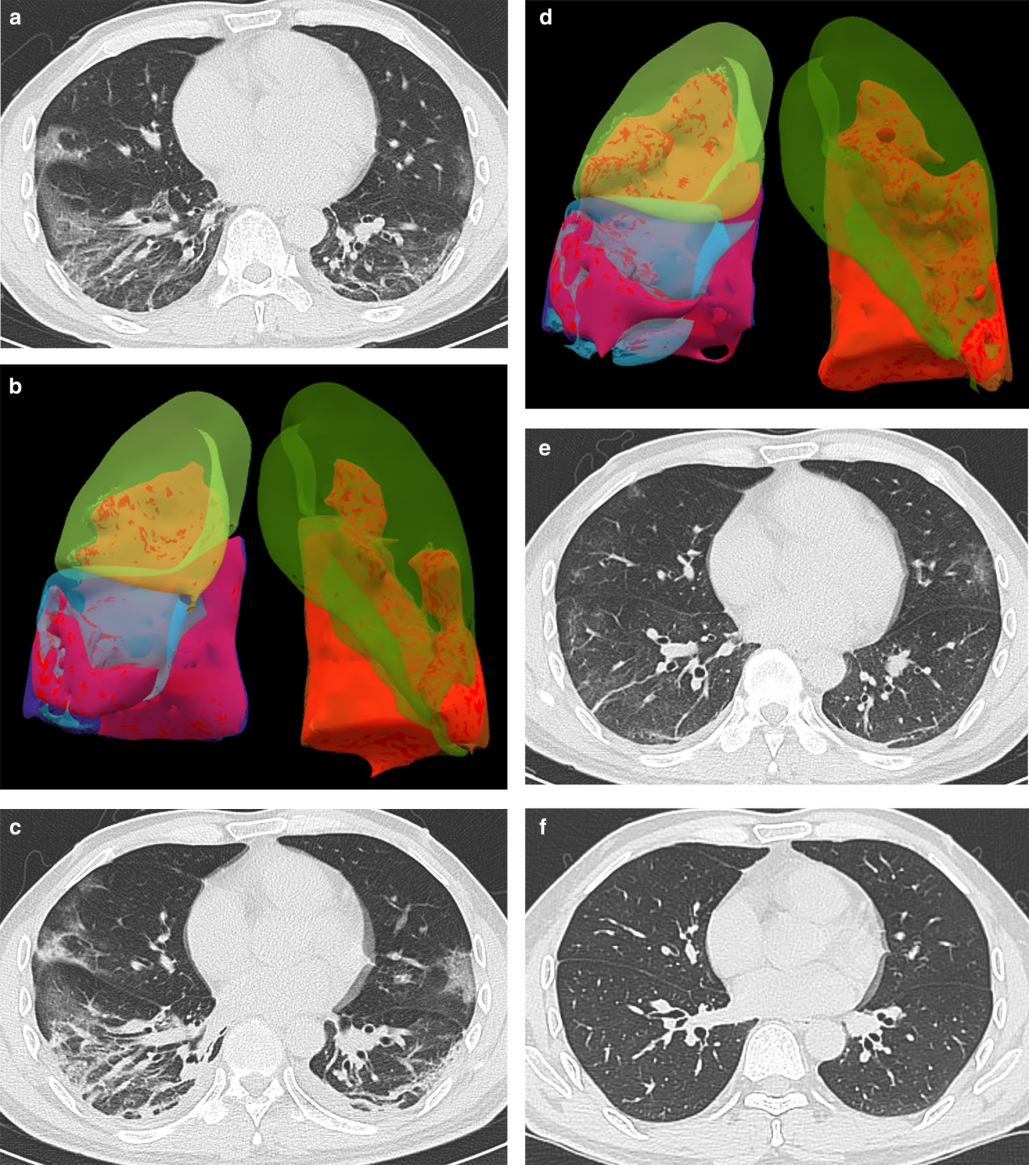
Male, 45-year-old, COVID-19. **a** Axial CT and (**b**) Volume Render (VR) Imaging. The initial CT and VR imaging obtained 7 days from onset of symptoms. The CT shows the patchy ground-glass opacity, crazy-paving pattern, irregular lines, and consolidation around the subpleural area, and pleura thickening. The VR imaging shows the lesions involve the bilateral lungs. **c** Axial CT and (**d**) VR Imaging. The peak CT and VR imaging obtained 3 days later. The CT shows that ground-glass opacity decrease, consolidation irregular lines, and pleural thickening increase, and bilateral pleura effusion occurred. The VR imaging shows the lesion size significantly increases.** e** Axial CT. The pre-discharge CT obtained 8 days later. The CT shows ground-glass opacity, irregular lines, and pleural thickening adhesions remain.** f** Axial CT. The follow-up CT obtained 34 days later shows a normal chest CT

**Fig. 5:**
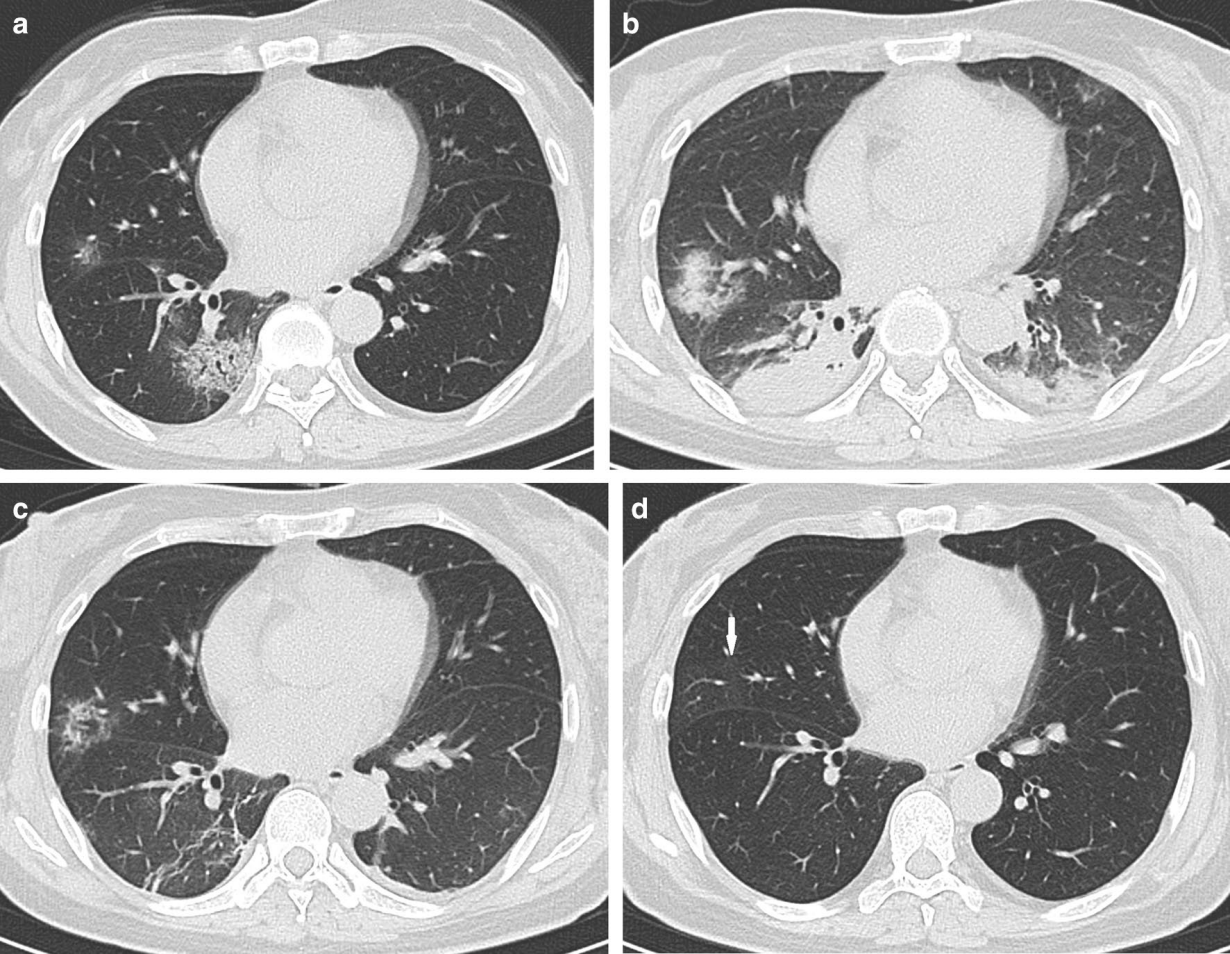
Female, 55-year-old, COVID-19. **a** The initial CT obtained 5 days from onset of symptoms shows the round ground-glass opacity (arrow) around the subplerual area of the left lower lobe. **b** The peak CT obtained 9 days later shows the large patchy consolidations and pleural thickening increase much more and air bronchograms are observed. **c** The pre-discharge CT obtained 8 days later shows consolidation, ground-glass opacity, irregular lines, reticulation, and pleura thickening. **d** The follow-up CT obtained 45 days later shows a few of ground-glass opacity remained in right middle lobe (arrow)

All 69 cases showed mainly distributing along the subpleural area on the initial, peak, and pre-discharge CTs, and nearly 1/3 of cases accompanying by distribution around peribronchivascular bundle (Table 1). The proportion of involvement in the lower lobes was slightly higher than that in the upper and middle lobes (*p* = 0.453–0.947) (Table 1) (Figs. 3, 4, 5).

**Table 1:** Distribution of initial, peak, and pre-discharge CTs of COVID-19 during hospitalization

	Initial CT (%)	Peak CT (%)	Pre-discharge CT (%)	*p* value
No	67^a^	69	69	
Subpleural distribution	45 (67.2)	47 (68.1)	46 (66.7)	0.983
Mixed distribution^b^	22 (32.8)	22 (31.9)	23 (33.3)	
Right lung				
Upper lobe	44 (65.7)	50 (72.5)	47 (68.1)	0.687
Middle lobe	38 (56.7)	43 (62.3	41 (59.4)	0.801
Lower lobe	57 (85.1)	60 (87.0)	59 (85.5)	0.947
Left lung				
Upper lobe	45 (67.2)	53 (76.8)	49 (71.7)	0.453
Lower lobe	56 (83.6)	61 (88.4)	60 (87.0)	0.703

^a^Two of 69 patients show no COVD-19 on CT

^b^Mixed distribution = distribution of subpleural and bronchovascular bandle

symptoms shows the round ground-glass

### Correlation between scores and intervals

**Fig. 6:**
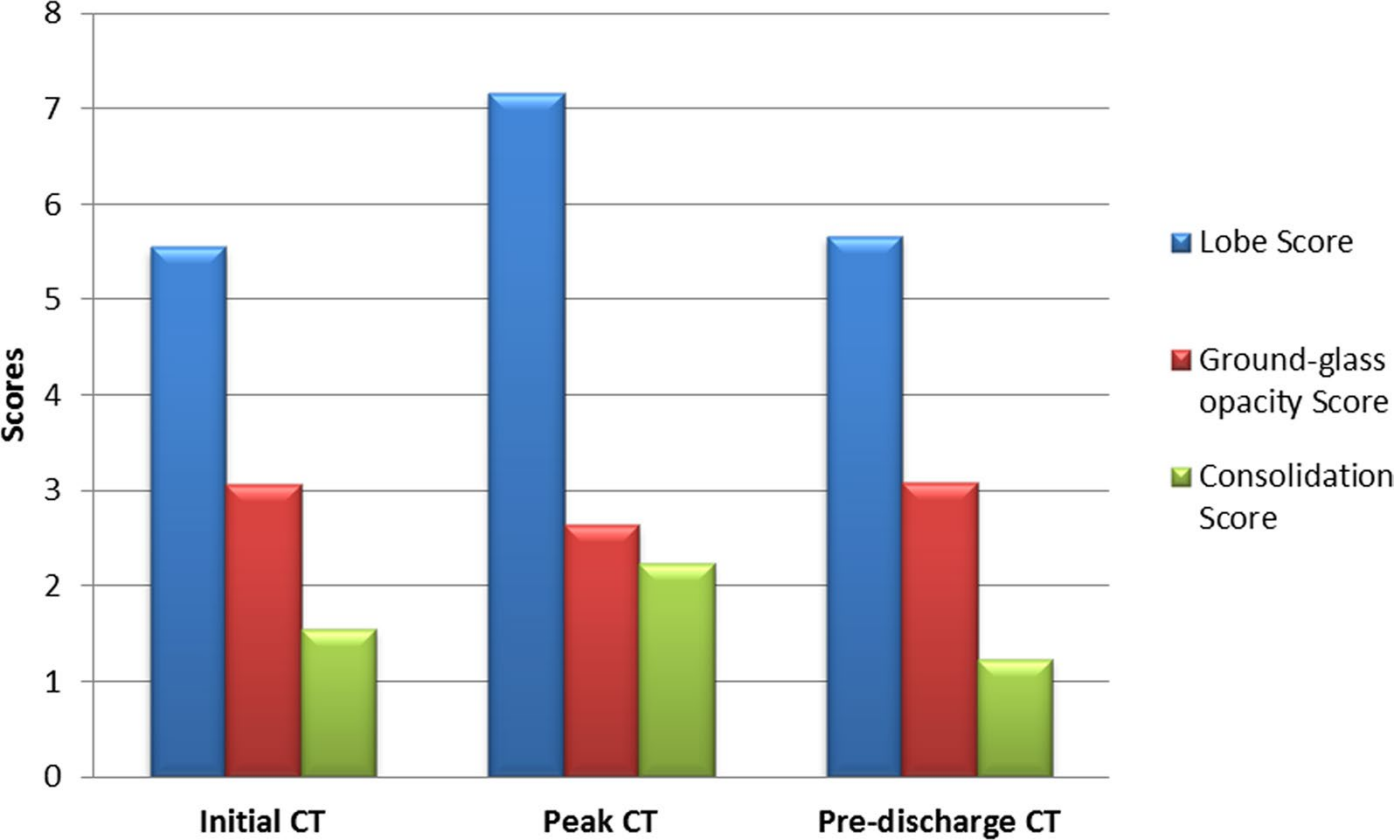
The lobe score, ground-glass opacity score, and consolidation score on initial CT, peak CT, and pre-discharge CT

The lobe score on peak CT (7.17 ± 4.762) was markedly higher than that of initial CT (5.55 ± 3.974, *p* < 0.001) and pre-discharge CT (5.67 ± 3.563, *p* < 0.001) (Fig. 6).

Lobe scores showed positive correlations with the intervals (*r* range, 0.301–0.669, *p* < 0.001) except the peak-initial interval (*p* = 0.210–0.400) (Table 2).

**Table 2:** Correlation coefficient between intervals and score of lobes percentage on initial, peak, and pre-discharge CTs of COVID-19 during hospitalization

	Initial CT (*p* value)	Peak CT (*p* value)	Pre-discharge CT (*p* value)
Initial interval	0.621 (< 0.001)	0.417 (< 0.001)	0.419 (< 0.001)
Peak interval	0.465 (< 0.001)	0.530 (< 0.001)	0.488 (< 0.001))
Pre-discharge interval	0.503 (< 0.001)	0.669 (< 0.001)	0.496 (< 0.001)
Peak-Initial interval	− 0.108 (0.372)	0.153 (0.210)	0.103 (0.400)
Pre-discharge-peak interval	0.320 (0.008)	0.482 (< 0.001)	0.301 (0.006)

### CT appearances

**Fig. 7:**
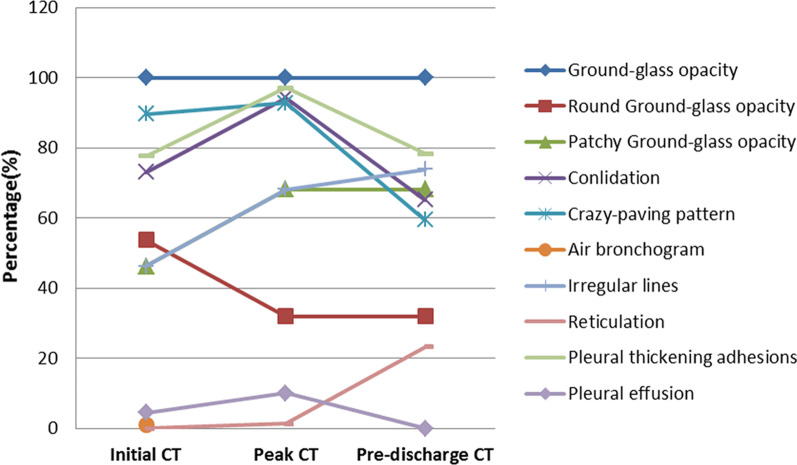
The CT appearences on the initial CT, peak CT, and pre-discharge CT

All 69 patients displayed GGO on the initial, peak, and pre-discharge CTs. Round GGOs on the initial CT (53.7%) more commonly observed than that on both of peak CT (31.9%, *p* = 0.010) and pre-discharge CT (31.9%, *p* = 0. 010) (Table 3) (Figs. 3, 7).

**Table 3:** Comparison among imaging findings of initial, peak, and pre-discharge CTs of COVID-19 during hospitalization

	Initial CT (%)	Peak CT (%)	Pre-discharge CT (%)	*p* value
No	67^a^	69	69	
Ground-glass opacity(GGO)	67 (100.0)	69 (100.0)	69 (100.0)	
Round GGO	36 (53.7)	22 (31.9)	22 (31.9)	0.011
Patchy GGO	31 (46.3)	47 (68.1)	47 (68.1)	
Consolidation	49 (73.1)	65 (94.2)	45 (65.2)	< 0.001
Crazy-paving pattern	53 (79.1)	59 (85.5)	34 (49.3)	< 0.001
Air bronchogram	60 (89.6)	64 (92.8)	41 (59.4)	< 0.001
Irregular lines	31 (46.3)	47 (68.1)	51 (73.9)	0.002
Only reticulation	0 (0.0)	1 (1.4)	16 (23.2)	< 0.001
Pleural thickening adhesions	52 (77.6)	67 (97.1)	54 (78.3)	0.002
Pleural effusion	3 (4.5)	7 (10.1)	0 (0)	0.015

^a^Two of 69 patients show no COVD-19 on CT

Consolidations on peak CT (94.2%) were more common than those on initial CT (73.1%, *p* = 0.006) and pre-discharge CT (65.2%, *p* < 0.001) (Figs. 3, 4, 5, 7). 49.3% of the pre-discharge CT displayed the crazy-paving pattern, significantly fewer than the initial CT (79.1%, *p* < 0.001) and peak CTs (85.5%, *p* < 0.001). Air bronchograms (59.4%) were less commonly found on pre-discharge CT than on initial (89.6%, *p* < 0.001) and peak CTs (92.8%, *p* < 0.001). Irregular lines on initial CT (46.3%) were less evident than those on peak (68.1%, *p* = 0.010) and pre-discharge CTs (73.9%, *p* = 0.001). Reticulations appeared on 23.2% of pre-discharge CT, more common than that on peak CT (1.4%, *p* < 0.001), and absent on initial CT (Figs. 3, 5, 7). Pleural thickenings were more commonly found on peak CT (97.1%) than initial CT (77.6%, *p* = 0.001) and pre-discharge CT (78.3%, *p* = 0.001) (Figs. 3, 4, 5). The pleural effusions were more commonly seen on peak CT (10.1%) than on initial CT (4.5%, *p* = 0.349), and absent on pre-discharge CT (*p* = 0.020) (Table 3) (Figs. 4, 7).

Other imaging findings included nodules in seven cases, but none lymphadenopathy.

### Comparing imaging manifestations of intervals

There were different manifestations in the initial CT, peak CT, and pre-discharge CT (Fig. 7). When consolidations, air bronchograms, and irregular lines were found on the initial CT and irregular lines on the pre-discharge CT, the initial intervals were much more extended than no these findings on the initial and pre-discharge CTs. When irregular lines were observed on the peak CT and irregular lines and air bronchograms on the pre-discharge CT, the peak intervals were more extended (Table 4). The pre-discharge intervals were more extended when irregular lines were found on peak CT and reticulations on pre-discharge CT, while the pre-discharge intervals were more shortened as consolidation and crazy-paving pattern on pre-discharge CT (Table 4).

**Table 4:** Comparison of intervals on initial, peak, and pre-discharge CTs of COVID-19^a^ during hospitalization

	Initial CT (yes^b^, days; no^c^, days; *p* value)	Peak CT (yes^b^, days; no^c^, days; *p* value)	Pre-discharge CT (yes^b^, days; no^c^, days; *p* value)
Initial Interval	Consolidation (6.65 ± 3.649; 3.78 ± 1.957; 0.002) Air bronchogram (6.23 ± 3.524; 2.86 ± 1.215; 0.015) Irregular line (7.29 ± 3.934; 4.67 ± 2.586; 0.002)	No significant statistically difference	Irregular line (6.31 ± 3.603; 4.17 ± 2.875; 0.026)
Peak Interval	No significant statistically difference	Irregular line (10.50 ± 3.443; 8.17 ± 3.892; 0.014)	Irregular line (10.37 ± 3.605; 7.89 ± 3.579; 0.014) Air bronchogram (10.54 ± 3.641; 7.89 ± 3.579; 0.014)
Pre-discharge Interval	No significant statistically difference	Irregular line (23.02 ± 6.191; 17.13 ± 8.374; 0.002)	Consolidation (19.53 ± 6.768; 23.92 ± 8.032; 0.019) Crazy-paving pattern (19.26 ± 6.072; 22.80 ± 8.345; 0.049) Reticulation (25.13 ± 7.356; 19.83 ± 7.130; 0.012)
Peak-initial Interval	Consolidation (3.22 ± 3.423; 5.78 ± 2.211; 0.004) Pleural thickening (3.40 ± 3.339; 5.67 ± 2.717; 0.019)	No significant statistically difference	No significant statistically difference
Pre-discharge-peak Interval	Crazy-paving pattern (12.26 ± 6.048; 8.50 ± 6.334; 0.044)	Irregular line (12.54 ± 5.995; 8.96 ± 6.116; 0.023)	Consolidation (9.80 ± 5.814; 14.25 ± 6.045; 0.004) Crazy-paving pattern (9.44 ± 5.177; 13.20 ± 6.663; 0.011) Reticulation (14.25 ± 6.445; 10.47 ± 5.947;0.032)

^a^The other imaging findings not shown in the table had no significantly statistically difference in intervals

^b^Yes = imaging manifestation

^c^No = no imaging manifestation

The peak-initial intervals were more shortened when consolidations and pleural thickenings on the initial CT (Fig. 4). The pre-discharge-peak intervals were more extended as crazy-paving pattern on the initial CT, irregular lines on the peak CT, and reticulations on the pre-discharge CT, but shortened as consolidation and crazy-paving pattern on pre-discharge CT (Table 4).

### Follow-up

Among 69 patients, 16 patients (23.2%) did not come to hospital for checks, and the other 53 patients (76.8%) were followed up. The 53 patients included 22 females and 31 males, and mean age 49.509 ± 14.780 years (range, 19–80 years). Median follow-up intervals were 60.0 (45.0, 89.5) days (range, 30–329 days). And there were 20 patients (37.7%) in absorption group (8 females and 12 males, and mean age 43.050 ± 11.450 years; range, 29-76 years; median follow-up interval, 68.0 (46.5, 109.8) days ) (Figs. 3, 4) and 33 patients (62.3%) in residual group (14 females and 19 males, and mean age 53.424 ± 15.337 years; range, 19-80 years; median follow-up interval, 56.0 (44.5,83.0) days) (Fig. 5), respectively. Between the two groups, the gender and follow-up interval had no significantly statistical difference, but the age of the residual group was elder than that of the absorption group (*p* = 0.012).

Residual lesions showed mainly distributing along the subpleural area on CT (90.9%, 30/33), and only 9.1% (3/33) of cases distributing along subpleural area and around peribronchivascular bundle, which showed no significantly statistical difference with initial CT, peak CT, and pre-charge CT, respectively (Fig. 5).

GGO (100.0%, 33/33) was still the most common CT pattern (Fig. 5), following irregular lines (33.3%, 11/33), reticulation (15.2%, 5/33), consolidation (6.1%, 2/33) and pleural thickening adhesion (6.1%, 2/33). Crazy-paving pattern, air bronchogram, and pleural effusion were not found. All the GGO presented as patchy GGO, not round GGO.

GGO morphology, consolidation, crazy-paving pattern, air bronchogram, and pleural thickening adhesions on follow-up CT showed significantly statistical difference with initial CT, peak CT, and pre-charge CT, respectively (*p* < 0.001). Irregular lines (33.3%) were less common than those on peak CT (68.1%, *p* < 0.001) and pre-discharge CT (73.9%, *p* = 0.001), but not initial CT (46.3%, *p* = 0.218). Reticulations (15.2%) were more common than those on initial CT (0.0%, *p* = 0.003) and peak CT (1.4%, *p* = 0.013), respectively, but not pre-charge CT (23.2%, *p* = 0.348). Pleural effusion (0.0%) had no significantly statistical difference with initial CT (4.5%, *p* = 0.549), peak CT (10.1%, *p* = 0.093), but pre-charge CT (0.0%, *p* value was not applicable).

## Discussion

COVID-19 showed the typical appearances of viral pneumonia on CT, with rapid progress and obvious changes in a short time. Through our study, we found that, first, the most progression pattern was type II (65.2%), and peak-initial interval was shorter than the pre-discharge-peak interval. Second, lobe score showed a positive correlation with intervals except peak-initial interval. Third, it was most commonly observed to round GGOs on initial CT, consolidations on peak CT, and reticulations on pre-discharge CT (Table 3) (Figs. 3, 4, 5, 7). Fourth, the initial intervals were much more extended when consolidation, air bronchogram, and irregular lines on initial CT (Fig. 4). The intervals were more extended as irregular lines and reticulation on CT (Table 4). Fifth, the viral pneumonia can be remained or completely absorbed on CT with follow-up (Figs. 3, 4, 5).

So far this century, there have been three serious infectious diseases caused by coronavirus, one of which was Severe Acute Respiratory Syndrome (SARS) that broke out in 2003 in China and spread to the whole world [12]; the second time was Middle East Respiratory Syndrome (MERS) in Iran (in 2012) [13]; the third time was COVID-19, which was first reported to the WHO Country Office in China on 31 December, 2019, and became a global event [1, 2]. COVID-19 can cause severe respiratory system Diseases. These infectious diseases caused by coronaviruses showed similar CT manifestations. Although part of their imaging findings superimposed, they also have their specific characteristics, especially in the dynamic changes of CT. Studies of SARS report that the peak pattern (70.3%) is the most common type, following by the fluctuating (17.4%), improving (7.3%), and deteriorating patterns (5.1%) [14]. In MERS cases, the most common type is the deteriorating pattern (53.3%), following by the peak (40.0%), fluctuating (6.7%), and improving patterns (0%) [15]. Here, we found that the most common type in our study was the peak pattern (65.2%), similar to SARS. But the improving pattern (33.3%) was significantly higher than that of SARS and MERS and the deteriorating pattern (1.5%) was significantly lower than that of MERS and slightly lower than that of SARS. Fluctuating pattern were observed in 17.4% of SARS and 6.7% of MERS, but absent in our study. The peak pattern was the dominate type in SARS and COVID-19 while the deteriorating pattern was the most common type in MERS [14, 15]. The deteriorating pattern was the least common type, which might be due to all of our cases were from discharged patients. It was special to have none fluctuating pattern in this study, the reason of which was not clear. However, the two studies are based on chest radiographs that are overlay imaging, so some detail changes cannot be clearly found. Our study was based on CT that can exhibit much more clearly lesions in the lungs than chest radiographs. Therefore, our study could accurately present the progression of the disease than those two studies. In this study, the peak-initial interval (7.65 ± 6.203 days) was much shorter than pre-discharge-peak interval (11.35 ± 6.228 days) (Fig. 2). Accordingly, COVID-19 progressed rapidly to the peak period, and then took longer time to gradually resolve until discharge.

Lobe score of COVID-19 exhibited positively correlated to most of intervals but not to peak-initial interval. That was, the larger the lesion sizes were associated the longer intervals (Table 2). As the consolidation and pleural thickening on the initial CT emerged, the peak-initial interval shortened, the disease progressed more rapidly. The round GGO was characteristic on the initial CT (Table 4, Fig. 4). When the irregular lines appeared on peak CT, indicating the occurrence of fibrotic changes, the peak, pre-discharge, and pre-discharge-peak intervals were extended. The intervals were also extended when the reticulations appeared on the CTs (Table 4). Therefore, when fibrosis manifestations (irregular lines and reticulation) occurred on CTs, intervals would be extended.

The imaging manifestations are based on the pathology that has been report for SARS and MERS [12, 13, 16, 17]. Moreover, it has been proposed that the pathology of COVID-19 is similar to that of SARS and MERS [18, 19]. In the early stage, COVID-19 shows that the exudation and proliferation of acute lung injury manifesting as edema, infiltration of inflammatory cells, proliferation of Type II alveolar cells and fibroblasts, protein exudation, and vascular congestion [19]. In the progression stage, the pathology shows diffuse alveolar injury, exfoliated alveolar cells with fibromucous secretion, pulmonary edema, hyaline membrane formation, monocytes infiltration (mainly lymphocytes) in alveolar cell septa, marked protein and fibrin exudates in the lung parenchyma, diffuse thickening of the alveolar wall, and fibroblasts and Type II alveolar cell hyperplasia, while focal fibroblast embolism can be seen in the alveolar cavity, presenting as acute respiratory distress syndrome [18, 20]. Then the most common manifestation of COVID-19 was GGO (Figs. 3, 4, 5, 7), which might be associated with alveolar edema, inflammatory cells infiltration, and thickening of the alveolar septum. Consolidation might be caused by alveolar edema, inflammatory cells, protein and cellulose-like mucus in the alveolar cavity. The crazy-paving pattern involved both parenchyma and interstitium. Both irregular lines and reticulation might represent the fibrotic changes, diffuse thickening of the alveolar wall, fibroblasts proliferation, thickening of alveolar septa, and the thickening intralobular lines. Pleural thickening and adhesion were also common imaging manifestations, which might be related to the infiltration of inflammatory cells in the alveolar septum, and fibroblasts [11, 18–20].

With follow-up in this study, the pulmonary lesions could be remained (62.3%) or completely absorbed (37.7%). And the ages of the patients in residual lesions group were elder than that of the patients in absorption group, which is in accord with the literature [21]. The most common CT pattern with follow-up was GGO, following irregular lines, and reticulation, which were similar to the literature [21, 22] (Fig. 5).

This study had some limitation. First, COVID-19 was an emerging infectious disease. The sample size was not large, but it could still indicate the imaging manifestations and dynamic changes of COVID-19. Second, the study mainly studied the dynamic changes of COVID-19 on chest CT, as well as the correlation analysis between imaging performances and time interval. There were fewer data on clinical features in the present study. However, there are some studies on the correlation of clinical characteristics of COVID-19 in the literatures [21–23]. For example, GGO and consolidation are the main manifestations in the acute stage of the disease. With the progress of the disease, some patients show the lesions are completely absorbed, and some patients show residual fibrosis lesions. It has been reported in the literature that the diffusion function of patients is decreased in the acute stage, and the abnormal diffusion function will still appear when the residual fibrosis changes in the lung in the recovery stage [7–10, 21, 22, 24]. In the future research, we will focus on the correlation between clinical features and CT imaging changes. Third, whether the interstitial lesions could be completely absorbed or not needed more long-term follow-up observation.

## Conclusions

In brief, COVID-19 can rapidly progress to the peak and gradually recovers. The most common pattern of progression was the peak pattern, following by improvement pattern and deterioration pattern. The intervals were closely related to lobe scores and CT appearances. The higher the lobe score, the longer the intervals. If consolidations, air bronchograms, and irregular lines on initial CT increase, the initial intervals will be shorter. If the irregular lines and reticulations appear on the peak CT and pre-discharge CT, the intervals will be extended. After follow-up, COVID-19 pneumonia can be completely absorbed while GGO, irregular lines, and reticulation also can be remained.

## Data Availability

The datasets used and/or analysed during the current study are available from the corresponding author on reasonable request.

## Abbreviations

COVID-19: Coronavirus disease 2019
CT: Computed tomography
GGO: Ground-glass opacity
Lobe Score: Percentage of lesions in one lobe
MERS: Middle east respiratory syndrome
RT-PCR: Fluorescence-based real-time reverse transcription-polymerase chain reaction
SARS: Severe acute respiratory syndrome
SARS-CoV-2: Severe acute respiratory syndrome-associated coronavirus 2
WHO: World Health Organization
